# Tracking the evolutionary footprint of Mpox in West Africa: phylogenetic and clade analysis

**DOI:** 10.1017/S0950268825100411

**Published:** 2025-10-07

**Authors:** Elijah Kolawole Oladipo, Stephen Feranmi Adeyemo, James Akinwumi Ogunniran, Possible Okikiola Popoola, Victoria Ajike Alabi, Joshua Opanike

**Affiliations:** 1 Division of Genome and Molecular Sciences, Helix Biogen Institute, Ogbomoso, Nigeria; 2 Division of Vaccine and Pharmacotherapies Design and Development, Helix Biogen Institute, Ogbomoso, Nigeria; 3 Department of Microbiology, Laboratory of Molecular Biology, Immunology and Bioinformatics, Adeleke University, Ede, Nigeria; 4 Department of Chemical Engineering, University of Birmingham, Birmingham, UK

**Keywords:** evolutionary, genomic surveillance, geographic zones, mpox, phylogenetic, West Africa

## Abstract

Mpox (formerly called monkeypox) is a zoonotic viral disease caused by the monkeypox virus (MPXV) that has recently emerged as a notable global health issue by spreading beyond its typical geographical zones in Central and West Africa. In this study, we conducted a phylogenetic and evolutionary investigation of MPXV in West Africa. We focussed on 167 complete genome sequences collected from human infections in Nigeria and Cameroon between 2019 and 2024, all of which were retrieved from the GSAID database. To analyse these sequences, we employed multiple sequence alignment using fast Fourier transform (MAFFT) and maximum likelihood techniques to identify conserved genomic variants and trace evolutionary patterns within the virus. Our findings revealed that all the MPXV strains studied belong to clade II, which is further subdivided into two subclades. Notably, this study documents the presence of two distinct subclades IIa and IIb, reflecting the complex and ongoing evolution of the virus in the region. The phylogenetic analysis reveals rapid mutations and suggests that MPXV is being transmitted from multiple lineages between Nigeria and Cameroon. This demands the need to further strengthen the surveillance and containment efforts in West Africa. This study highlights the role of genomic surveillance in monitoring the evolution and spread of the MPXV, particularly in regions with limited available data.

## Introduction

The monkeypox virus (MPXV) is an enveloped double-stranded DNA virus that belongs to the *Orthopoxvirus* genus of the *Poxviridae* family and the *Chordopoxvirinae* subfamily [1, 2]. In recent years, monkeypox (Mpox), a zoonotic disease caused by the MPXV, has transcended its primary association with Central and West Africa, emerging as a notable global public health concern [3]. Two genetic clades of the MPXV exist, clade I and clade II, which include subclades Ia and Ib, and subclades IIa and IIb [4].

Mpox is a viral zoonotic reemerging disease resulting in smallpox-like human diseases [5]. Human MPXV infections historically arise from animal-to-human transmission, and various animal species are susceptible to MPXV infections, including a range of rodents and nonhuman primates [6]. Since the first human Mpox case was recorded in 1970 in the Democratic Republic of the Congo (DRC), Mpox has been identified in many other countries in Africa and around the world [7].

Based on the genetic and geographical location first detected, there are two recognized phylogenetic branches, i.e. the Central African (Congo Basin) clade and the West African clade [5]. According to the latest nomenclature, the former Congo Basin (Central African) clade is clade I, subdivided into Ia and Ib, and the former West African clade is clade II, which was further subdivided into IIa and IIb [4]. Clade IIb, in particular, is associated with the 2022–2024 multicountry outbreaks and has undergone rapid diversification, leading to the emergence of multiple sublineages characterized by genomic mutations. These include lineages such as IIb A, IIb A.1, IIb A.1.1, IIb A.2, IIb A.2.1, IIb A.2.2, IIb A.2.3, IIb A.3, and IIb B.1, many of which have been implicated in sustained human-to-human transmission outside Africa. Notably, IIb B.1 was the dominant lineage in the 2022 global outbreak, first identified in non-endemic countries such as the United Kingdom, Spain, and the United States [8, 9]. The emergence of these sublineages reflects the virus’s adaptive response to new epidemiological settings and highlights the importance of continuous genomic surveillance.

Recent Mpox outbreaks around the world highlight the importance of improved universal health care systems. The unpredictable and widespread nature of the MPXV indicates the shortcomings of current surveillance systems. Considering the complex impact of MPXV infections on pregnant women, children, and immunosuppressed individuals, these clusters of population need to be included in the disease surveillance system. Based on the recent Mpox outbreaks, the number of cases tends to be higher in individuals aged 26–40 [10].

Prior to 2017, Mpox cases were primarily confined to endemic regions of Central Africa (clade I) and West Africa (clade II, predominantly IIa), with no evidence of a globally disseminated clade IIb epidemic. During this period, most human infections resulted from zoonotic spillovers from sylvatic reservoirs, while human-to-human transmission was rare and usually limited to short transmission chains [2, 11, 12]. Although occasional cases were reported outside Africa, such as the 2003 US outbreak linked to imported rodents, there were no sustained international outbreaks until the 2022–2024 multicountry events driven by clade IIb [4, 8]. Historically, Mpox has been primarily associated with zoonotic transmission from a wide range of susceptible animals, including rodents and nonhuman primates [6]. Since the first recognized human case in 1970 in the DRC, Mpox has been detected in multiple countries across Central and West Africa, with sporadic imported cases recorded in recent decades [13]. Clinical manifestations resemble those of smallpox, and higher case fatality ratios (CFRs) of up to 11% have been reported historically for clade I infections among unvaccinated individuals. In contrast, the CFR in the 2022–2024 clade IIb outbreaks has been notably lower [4, 14].

The MPXV, being a DNA virus, has slower rates of evolution and higher genetic stability compared to other pathogens [15, 16], which suggests that it is less probable for major changes in the modus operandi of infection and transmission by this virus. While the epidemiological data showed a high rate of spread of clade Ib in East Africa, this could be explained by the highly developed transportation network that allows individuals to travel long distances in short periods, facilitating cross-border migration, which likely contributed to rapid virus dissemination [17].

A recent study by Oladipo et al. (2024) provides a new strategy for the treatment of *Poxviridae* infections. Three genes, namely E8L, E7R and H3L, associated with virus attachment and virulence were chosen based on the genome of vaccinia virus and MPXV for the development of a candidate mRNA vaccine against vaccinia virus and MPXV infection. All the epitopes used for the vaccine candidate were generated using different bioinformatics tools. Then, additional components were incorporated to ensure stability and efficacy, such as 5′ cap, 5′ UTR (untranslated region), adjuvant sequence, 3′ UTR, poly(A) tail. These safety measures included testing for human homology (to avoid autoimmune responses) and *in silico* immune simulations (to mimic the immune response of the human host to the designed mRNA vaccine). The construct showed the characteristics of a prominent vaccine candidate and calls for an *in vivo* study to further validate its safety and efficacy [18].

The WHO closely monitors outbreaks and takes action by sharing information and coordinating with member states and partners to provide updates, guidance, and support. From 1 January 2022 through 30 April 2025, the WHO has received reports of approximately 142,151 laboratory-confirmed Mpox cases and 328 deaths, spanning 133 reporting countries across all six WHO regions. Meanwhile, the number of reported confirmed cases in 2024 and 2025 is approximately 19,117 and 17,193, respectively, resulting in about 125 deaths [19]. The recent increase in Mpox cases is changing because the human MPXV has already clearly emerged in the immunity gap left by the eradication of smallpox and cessation of smallpox vaccination, making knowledge and prevention critical [2]. Thus, its report, epidemiological study, and prevention are critical and timely.

## Methodology

### Nucleotide sequence retrieval and alignment tool

The complete genomes of human MPXV were retrieved from the Global Initiative on Sharing All Influenza Data (GISAID) database [20] on 24 August 2024. The sequences retrieved were those from countries in West Africa (Nigeria, Cameroon, and the DRC). The sequences were retrieved based on the country, date of data collection, and clade. They were then prepared for further analysis.

### Analysis of the retrieved sequences

The retrieved sequences were further analysed based on the available data. The datasets were organized based on the number of sequences available across the countries, the number of sequences available per year (2019–2024), the number of occurrences of each of the clades from West Africa, and the occurrence of the clades per country.

### Phylogenetic analysis

The phylogenetic tree was constructed by conducting a phylogenetic analysis of the retrieved sequences. This was constructed to determine the common ancestor of each strain using the MAFFT version 7 server (https://mafft.cbrc.jp/alignment/server/index.html) [21, 22].

### Conserved variants

A comparative analysis of strains within clades was carried out using the MAFFT bioinformatics tool based on statistical analysis, to determine positions in the genomic sequences and the reference sequence [23, 24, 25]. A closer phylogenetic analysis using two sequences from the respective countries and a representative from each of the clades (including the reference sequence) was conducted to further identify the similarities between the sequences, and the Geneious Prime server (https://www.geneious.com/) was used to analyse genome identity.

## Results

### Genome retrieval and analysis of the retrieved sequences

**Figure 1.**
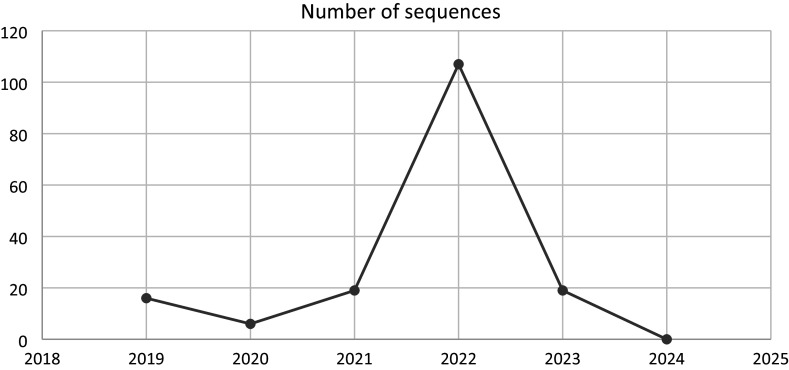
Number of sequences of the complete genome per year from West Africa, with the year 2022 housing the highest number of sequences (107).

**Figure 2.**
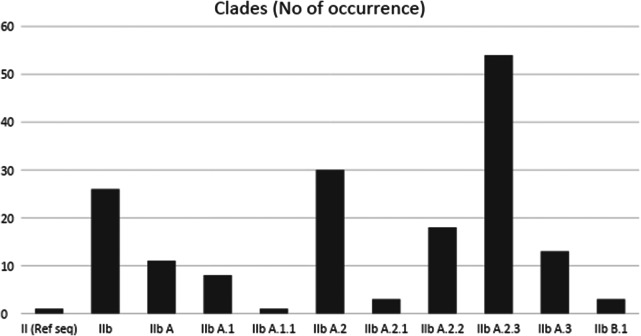
Number of occurrences of the lineages of clade IIb (from 2019 to 2024) in West Africa. Lineage IIb A.2.3 showed high occurrence.

**Figure 3.**
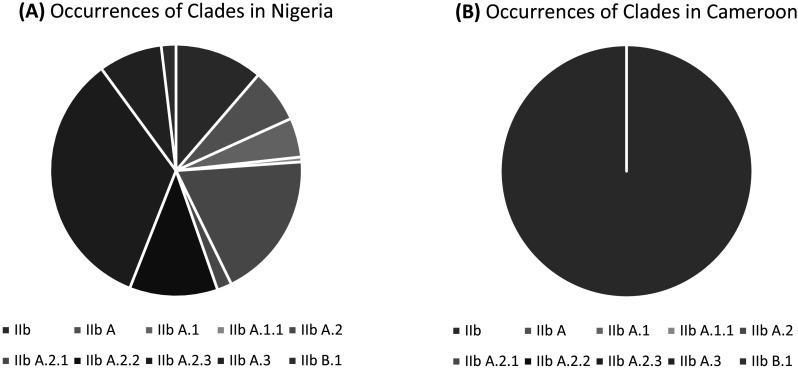
Chart showing the occurrences of the lineages of clade IIb per country. (A) Occurrences of the lineages of clade IIb in Nigeria; lineage IIb A.2.3 has the highest occurrence (54). (B) Occurrences of the lineages of clade IIb in Cameroon; clade IIb, with eight occurrences, is the only known clade.

The retrieved whole-genome sequences (WGSs) include 95% from Nigeria and 5% from Cameroon; there are no WGSs from other countries in West Africa. Further details are presented in Table 1. Figure 1 presents a chart showing the number of sequences available per year. Figure 2 shows the number of occurrences of each of the clades, while Figure 3 highlights the occurrences of the lineages of clade IIb per country.

**Table 1.** Total number of sequences present (including complete genomes and partial sequences) and the total number of complete genomes available in West Africa between 2019 and 2024

S/N	Countries	Total number of sequences present	Complete genome sequences (2019–2024)
1	Benin	4	0
2	Burkina Faso	0	0
3	Cameroon	17	8
4	Cape Verde	0	0
5	Cote d’Ivoire	0	0
6	Ghana	0	0
7	Guinea	0	0
8	Guinea Bissau	0	0
9	Liberia	2	0
10	Mali	0	0
11	Mauritania	0	0
12	Niger	0	0
13	Nigeria	177	159
14	Senegal	0	0
15	Sierra Leone	0	0
16	The Gambia	0	0
17	Togo	0	0

### Phylogenetic analysis

**Figure 4.**
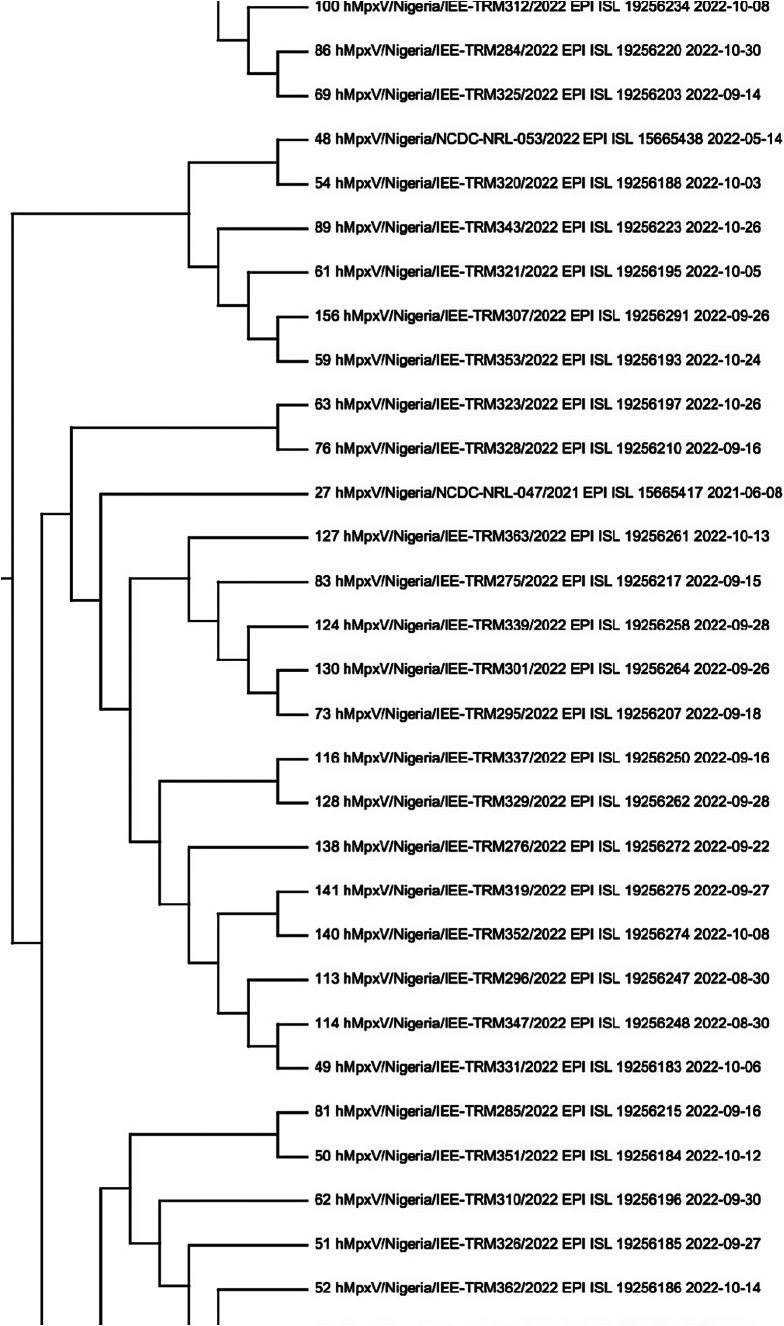
A part of the maximum likelihood tree of the retrieved 168 sequences (including the reference sequence) of human monkeypox virus across West Africa. Supplementary data 2: Complete maximum likelihood tree of the retrieved 168 sequences (including the reference sequence) of human monkeypox virus across West Africa. https://drive.google.com/file/d/1-qcV1_x-03oqXPv8mbd6vVU3Y7zKCYf1/view?usp=sharing.

**Figure 5.**
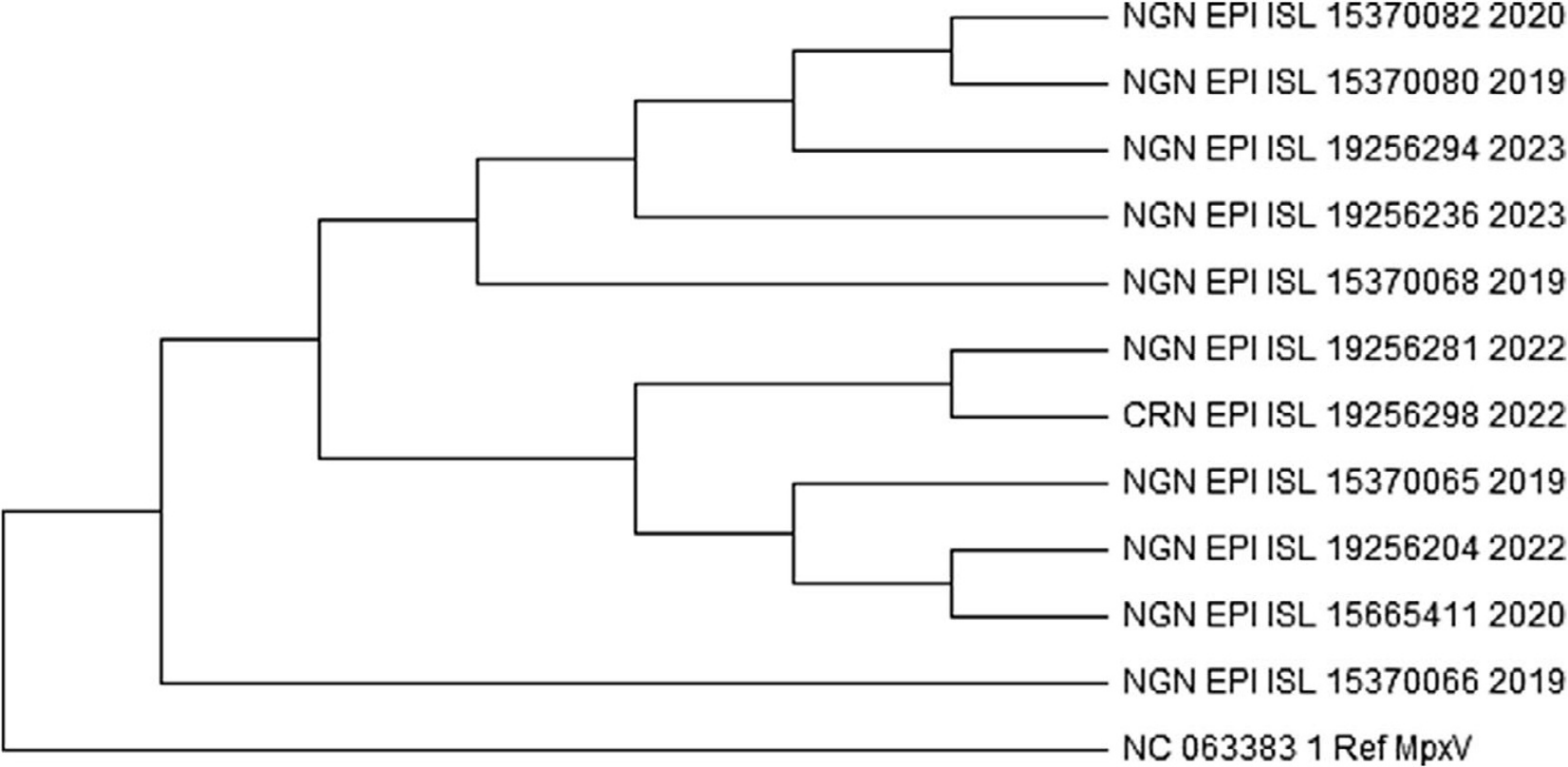
Maximum likelihood tree showing a closer phylogenetic analysis of human monkeypox virus from the selected sequences. The Nigerian sequence with the accession number EPI_ISL_19256281 and a Cameroon sequence with the accession number EPI_ISL_19256298 show a very close relationship.

The analysis involved 168 nucleotide sequences, including a reference sequence. Evolutionary history was analysed using the maximum likelihood method based on the general time reversible model. The bootstrap phylogenetic tree obtained from 1,000 replicates was taken to represent the evolutionary history of the sequences analysed. From the 168 sequences, only two sequences from Nigeria (accession number EPI_ISL_19256281 and EPI_ISL_19256276) shared a monophyletic group with the sequences from Cameroon as shown in Figure 4 and Supplementary data 2. On the other hand, a sequence with the accession number EPI_ISL_15665411 was found to be quite different from every other sequence. This further proves the exception of its clade (IIb A.1.1). A closer phylogenetic analysis (Figure 5) using two sequences from the monophyletic group, each from the respective countries, and a representative from each of the clades, including the reference sequence, further demonstrates the similarities and closeness of the sequences. The analysis conducted using Geneious Prime further proves that the sequences have 98.5% identical sites and 99.5% pairwise identity.

### Conserved variants

**Figure 6.**
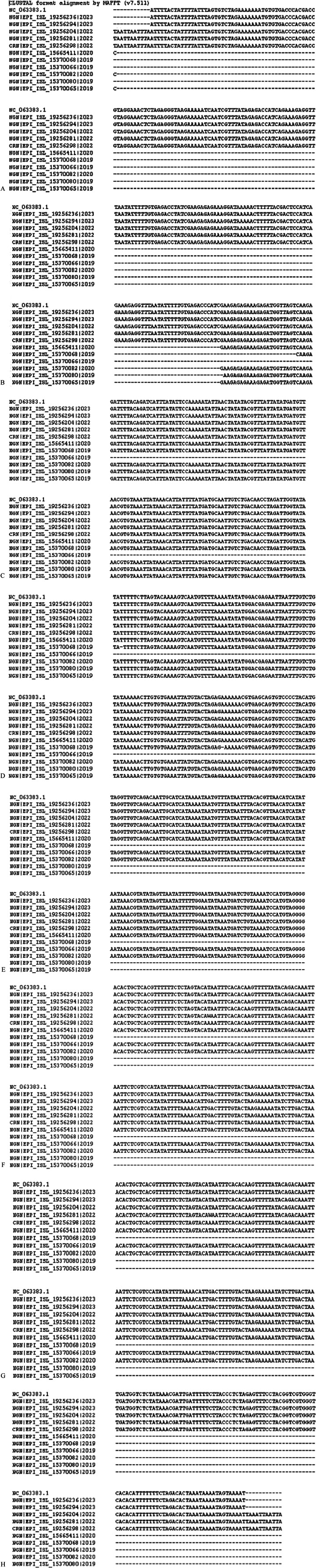
(A–G) Consensus variants between the ten strains further studied. Supplementary data 3: https://drive.google.com/file/d/1g33iTj9jVoj_awqgQvWKLUoiWcbvh41d/view?usp=drive_link

Figure 6 (A–G) and Supplementary data 3 show common features and differences between the lineages of the clades, including the reference lineages (IIb, IIb A, IIb A.1.1, IIb A.2, IIb A.2.1, IIb A.2.2, IIb A.2.3, IIb A.3, IIb B.1) from Nigeria and Cameroon, and the reference human MPXV (hMpoxV). Polymorphisms were detected by comparing alignment sequences to the consensus. The allele with a frequency higher than 50% is considered the consensus at each location. Without a 50% allele frequency, N is ambiguous.

## Discussion

Mpox gained global attention in 2022 due to a widespread outbreak in non-endemic regions, with transmission often linked to sexual contact. This sudden surge in Mpox cases and its unprecedented spread across non-endemic areas have led to public health emergencies. Two distinct genetic clades of the MPXV, which include clade I and clade II (both subdivided into Ia and Ib, and IIa and IIb, respectively), are now found globally [2]. The 2023–2024 Mpox outbreaks in East Africa [17] are linked to clade Ib MPXV.

Clade I, previously referred to as the Central African or Congo Basin clade, has been predominantly reported in Central African countries, particularly the DRC, where it has been associated with higher virulence and sustained human-to-human transmission [26, 27]. In contrast, clade II, formerly known as the West African clade, has historically circulated in West African nations, with Nigeria being a notable epicentre of recent resurgence since 2017. Cameroon is unique in that it is the only country confirmed to have recorded cases of both clade I and clade II viruses [28]. However, clade I cases from Cameroon have been sparse and date back to earlier outbreaks. These distinctions have important implications for understanding the evolutionary trajectory and public health risk of MPXV across Africa and beyond. Both clades are now found in all regions of the world, linked to ongoing outbreaks in Africa and around the world [29, 30]. Understanding the potential chains of transmission through sequence data provides deeper insights into the occurrence of mutations and the spread of infection.

The available sequences from West Africa during this period were primarily from Nigeria and Cameroon, highlighting the limited data completeness in the region as reported by the WHO [10]. The lack of genomic data hampers the understanding of potential chains of transmission and effectiveness of interventions and complicates control efforts. Therefore, it is essential to enhance the capacity of genomic analysis in the region through sustainable partnerships between the private and public sectors.

The 167 human Mpox isolate sequences from West Africa (Nigeria and Cameroon), retrieved from the GISAID database between 2019 and 2024, revealed that all strains belong to clade IIb. This result is consistent with the findings of Okwor et al. (2023) [31]. Furthermore, in this study, we came across nine lineages of IIb, namely IIb A, IIb A.1, IIb A.1.1, IIb A.2, IIb A.2.1, IIb A.2.2, IIb A.2.3, IIb A.3, and IIb B.1, between 2019 and 2024, highlighting the region’s evolving genomic landscape.

The predominance of clade IIb sequences in our dataset reflects the ongoing global spread and microevolution of the MPXV, particularly following the 2022 multicountry outbreak. Our analysis reaffirms that clade IIb has diversified into several lineages, being especially notable for its global dissemination and sustained human-to-human transmission. These lineages are defined by distinct mutational signatures and are thought to reflect adaptation to new transmission dynamics and host populations. The emergence of these lineages highlights the evolutionary plasticity of MPXV and underscores the need for high-resolution genomic surveillance to track lineage emergence, assess transmissibility, and monitor potential changes in pathogenicity or vaccine sensitivity. Our findings align with recent studies that show increasing genomic divergence within clade IIb [8, 9], underscoring the virus’s capacity for rapid adaptation in the human population.

Multiple sequence alignment separated the strains studied into distinct lineages depending on their divergence from their common ancestor. One of the isolates from Cameroon with accession number EPI_ISL_19256298 was in a monophyletic group shared with only two sequences from Nigeria with accession numbers EPI_ISL_19256281 and EPI_ISL_19256276. A comparative study was conducted on the strains from Nigeria, a monophyletic strain from Cameroon, a representative of the other clades, and the reference Mpox genome. Results from Geneious Prime revealed that all twelve sequences had 98.5% identical sites and 99.5% pairwise identity. The Cameroon isolate with accession number EPI_ISL_19256298 showed 99.9% identity with the Nigerian isolate EPI_ISL_19256281. All isolates also shared consensus similarity with the reference sequence NC_063383.1, indicating a common ancestor.

All of these sequences were retrieved in 2022, a period marked by the increased global circulation of clade IIb MPXV. While the phylogenetic clustering and geographical distribution of sequences may suggest the possibility of transboundary transmission, it is important to recognize that such patterns could also result from shared zoonotic reservoirs across borders or multiple, independent introductions into different regions. For instance, in border areas where wildlife habitats and human activities overlap, similar strains may emerge from parallel spillover events rather than direct human-to-human spread. Furthermore, the epidemiological context of 2022 includes reports of reverse importation into Nigeria from countries with confirmed outbreaks, complicating the interpretation of transmission directionality [32]. Therefore, distinguishing between sequences linked to the Nigerian or global outbreak of clade IIb and those potentially arising from localized zoonotic events is crucial for a more comprehensive understanding. The maximum branch length observed (0.00330, from sequence EPI_ISL_15665434) still indicates overall genetic proximity among the sequences, possibly reflecting either recent common ancestry or rapid yet constrained viral evolution. Overall, while cross-border transmission remains a plausible and critical factor to monitor, genomic and epidemiological data must be interpreted jointly to avoid overestimating their role without supporting field evidence.

From a genomic perspective, the minimal variation across most sequences, reflected in the short branch lengths in our phylogenetic analysis, suggests that the MPXV remains relatively stable, even as it spreads across borders. The marked conservation observed across the MPXV genomes analysed in this study has vital implications for public health preparedness and response. This high level of genetic stability suggests that diagnostic tools and therapeutic strategies developed based on conserved regions are more likely to remain effective across diverse strains and outbreaks. For example, viral surface and core proteins such as A29, A30, A35, and M1, known for their immunogenicity, have been identified as key targets in various *in silico* multi-epitope vaccine constructs, showing promise for broad cross-protection [18, 33]. These conserved sequences also aid in the refinement of molecular diagnostics, increasing detection reliability, especially in regions where sequencing capacity is limited [34]. Thus, the limited variation among circulating MPXV strains may significantly accelerate the development and deployment of vaccines, antivirals, and rapid diagnostic tools, ultimately supporting a more targeted and timely public health response.

## Limitations

While this study provides important insights into the evolutionary trends and spread of MPXV in West Africa, several limitations must be acknowledged.

First, a key challenge was the lack of accompanying epidemiological metadata, such as age, sex, and clinical outcomes, for the genomic sequences included in our analysis. This limitation, which also exists in the source database (GISAID), restricted our ability to explore demography-specific patterns of transmission and disease burden. For instance, although it is well established that children can be affected by clade II viruses, this dimension could not be adequately reflected in our study due to the absence of such data.

Second, the study is constrained by the unavailability of MPXV sequences from animal hosts in West Africa during the 2019–2024 period. No animal-derived clade II sequences were found in our dataset (maybe due to incomplete metadata) as of our retrieval date. This gap is significant, considering that the MPXV was historically assumed to be primarily zoonotic. The lack of animal genomic data makes it difficult to assess the current role of animal reservoirs and zoonotic spillovers, especially given that early public health responses underemphasized human-to-human transmission, particularly sexual transmission, which was only widely recognized after the 2022 global outbreak.

Additionally, this study is geographically limited to Nigeria and Cameroon, the only West African countries with high-quality MPXV sequences available in the GISAID EpiPox database at the time of data retrieval (24 August 2024). Notably, despite countries such as Ghana reporting more than 100 confirmed Mpox cases between 2022 and 2023, no publicly accessible genomic sequences from Ghana or several other affected West African nations were available in GISAID or GenBank during our data collection. This underrepresentation may reflect challenges in genomic surveillance infrastructure and reporting, and it underscores the need for more comprehensive regional data to better understand the evolution and transmission dynamics of the virus.

## Conclusion

Based on our phylogenetic and evolutionary analyses, the data support the evidence of sustained human-to-human transmission of MPXV within West Africa, particularly during the 2022 outbreak period. The sequences analysed, obtained from Nigeria and Cameroon, show strong genetic relatedness and suggest a common recent ancestor closely aligned with the MPXV human genome reference sequence from Nigeria. However, it is important to acknowledge that our dataset is geographically limited and does not include genomes from all West African countries; hence, broader conclusions about the origin of the outbreak across the entire region should be made cautiously. While our results may point to a single lineage introduction or a shared local zoonotic reservoir, definitive claims regarding the direction or source of introduction require further genomic and epidemiological investigation. These findings nonetheless provide valuable insights for continued biological study, clinical monitoring, and public health response to MPXV infections in West Africa.

## Supporting information

10.1017/S0950268825100411.sm001 Oladipo et al. supplementary material Oladipo et al. supplementary material

## Data Availability

All data generated or analysed during this study are included in this work and its supplementary information.

## Supplementary material

The supplementary material for this article can be found at http://doi.org/10.1017/S0950268825100411.
