# Supplementary material for: Tracking the evolutionary footprint of Mpox in West Africa: phylogenetic and clade analysis
Source: Epidemiol Infect. 2025 Oct 7;154:e46. doi: 10.1017/S0950268825100411 (PMC13100924; doi:10.1017/S0950268825100411)
Supplement: Oladipo et al. supplementary material [file S0950268825100411sup001.zip › S0950268825100411sup001.docx]

**Tracking the Evolutionary Footprint of Mpox in West Africa: Phylogenetic and Clade Analysis**

**Supplementary data 1:** Characteristics of the monkeypox virus complete genome from West Africa used in this study.

| **S/N** | **Country** | **Accession ID** | **Clade/ Lineage** | **Date of collection** | **Source** | **Age** | **Gender** |
| --- | --- | --- | --- | --- | --- | --- | --- |
| 1 | Reference | NC_063383.1 | Clade II | 08/2018 |  | - | - |
| 2 | Nigeria | EPI_ISL_15370065 | IIb A.1 | 1/16/2019 |  | - | - |
| 3 |  | EPI_ISL_15370066 | IIb A | 2/3/2019 |  | - | - |
| 4 |  | EPI_ISL_15370067 | IIb A | 2/26/2019 |  | - | - |
| 5 |  | EPI_ISL_15370068 | IIb A.3 | 3/14/2019 |  | - | - |
| 6 |  | EPI_ISL_15370069 | IIb A | 3/19/2019 |  | - | - |
| 7 |  | EPI_ISL_15370071 | IIb A | 4/3/2019 |  | - | - |
| 8 |  | EPI_ISL_15370072 | IIb A.1 | 4/29/2019 |  | - | - |
| 9 |  | EPI_ISL_15370073 | IIb A.1 | 4/29/2019 |  | - | - |
| 10 |  | EPI_ISL_15370074 | IIb A.1 | 4/29/2019 |  | - | - |
| 11 |  | EPI_ISL_15370075 | IIb A | 5/13/2019 |  | - | - |
| 12 |  | EPI_ISL_15370076 | IIb A.1 | 6/8/2019 |  | - | - |
| 13 |  | EPI_ISL_15370077 | IIb A.1 | 7/1/2019 |  | - | - |
| 14 |  | EPI_ISL_15370078 | IIb A.1 | 8/19/2019 |  | - | - |
| 15 |  | EPI_ISL_15370079 | IIb A.1 | 11/16/2019 |  | - | - |
| 16 |  | EPI_ISL_15370080 | IIb A.2 | 12/19/2019 |  | - | - |
| 17 |  | EPI_ISL_15370081 | IIb A.2 | 12/20/2019 |  | - | - |
| 18 |  | EPI_ISL_15370082 | IIb A.2.1 | 1/23/2020 |  | - | - |
| 19 |  | EPI_ISL_15665409 | IIb | 1/21/2020 |  | 35 | Male |
| 20 |  | EPI_ISL_15665410 | IIb A.3 | 2/10/2020 |  | - | Male |
| 21 |  | EPI_ISL_15665411 | IIb A.1.1 | 3/11/2020 |  | 20 | Male |
| 22 |  | EPI_ISL_15665412 | IIb | 10/12/2020 |  | 38 | Male |
| 23 |  | EPI_ISL_15665413 | IIb | 12/28/2020 |  | 33 | Male |
| 24 |  | EPI_ISL_15665414 | IIb A.2 | 3/15/2021 |  | 41 | Male |
| 25 |  | EPI_ISL_15665415 | IIb | 3/13/2021 |  | 21 | Female |
| 26 |  | EPI_ISL_15665416 | IIb | 5/26/2021 |  | 29 | Male |
| 27 |  | EPI_ISL_15665417 | IIb A.2 | 6/8/2021 |  | 50 | Male |
| 28 |  | EPI_ISL_15665418 | IIb | 8/18/2021 |  | 42 | Male |
| 29 |  | EPI_ISL_15665419 | IIb A.3 | 7/2/2021 |  | 20 | Female |
| 30 |  | EPI_ISL_15665420 | IIb | 7/16/2021 |  | 72 | Male |
| 31 |  | EPI_ISL_15665421 | IIb | 7/27/2021 |  | 29 | Male |
| 32 |  | EPI_ISL_15665422 | IIb | 7/28/2021 |  | 32 | Male |
| 33 |  | EPI_ISL_15665423 | IIb | 7/31/2021 |  | 32 | Male |
| 34 |  | EPI_ISL_15665424 | IIb A | 8/5/2021 |  | 32 | Male |
| 35 |  | EPI_ISL_15665425 | IIb | 10/10/2021 |  | 26 | Male |
| 36 |  | EPI_ISL_15665426 | IIb A | 11/17/2021 |  | 31 | Male |
| 37 |  | EPI_ISL_15665427 | IIb | 11/24/2021 |  | 29 | Male |
| 38 |  | EPI_ISL_15665428 | IIb | 12/1/2021 |  | 35 | Male |
| 39 |  | EPI_ISL_15665429 | IIb A | 11/8/2021 |  | 49 | Male |
| 40 |  | EPI_ISL_15665430 | IIb A.2.1 | 12/14/2021 |  | 37 | Male |
| 41 |  | EPI_ISL_15665431 | IIb | 12/27/2021 |  | 37 | Male |
| 42 |  | EPI_ISL_15665432 | IIb A | 2/10/2022 |  | 45 | Male |
| 43 |  | EPI_ISL_15665433 | IIb | 3/14/2022 |  | 33 | Male |
| 44 |  | EPI_ISL_15665434 | IIb | 4/3/2022 |  | 26 | Male |
| 45 |  | EPI_ISL_15665435 | IIb A.3 | 4/25/2022 |  | 45 | Male |
| 46 |  | EPI_ISL_15665436 | IIb A.3 | 5/5/2022 |  | 30 | Male |
| 47 |  | EPI_ISL_15665437 | IIb A.2.3 | 4/29/2022 |  | 31 | Male |
| 48 |  | EPI_ISL_15665438 | IIb A.2 | 5/14/2022 |  | 40 | Male |
| 49 |  | EPI_ISL_19256183 | IIb A.2 | 10/6/2022 |  | - | - |
| 50 |  | EPI_ISL_19256184 | IIb A.2 | 10/12/2022 |  | - | - |
| 51 |  | EPI_ISL_19256185 | IIb A.2.2 | 9/27/2022 |  | - | - |
| 52 |  | EPI_ISL_19256186 | IIb A.2.2 | 10/14/2022 |  | - | - |
| 53 |  | EPI_ISL_19256187 | IIb A.2.3 | 3/3/2023 |  | - | - |
| 54 |  | EPI_ISL_19256188 | IIb A.2 | 10/3/2022 |  | - | - |
| 55 |  | EPI_ISL_19256189 | IIb A.2.3 | 5/16/2023 |  | - | - |
| 56 |  | EPI_ISL_19256190 | IIb A.2.3 | 3/15/2023 |  | - | - |
| 57 |  | EPI_ISL_19256191 | IIb A.2.3 | 3/3/2023 |  | - | - |
| 58 |  | EPI_ISL_19256192 | IIb A.2.3 | 9/27/2022 |  | - | - |
| 59 |  | EPI_ISL_19256193 | IIb A.2 | 10/24/2022 |  | - | - |
| 60 |  | EPI_ISL_19256194 | IIb A.2.3 | 9/12/2022 |  | - | - |
| 61 |  | EPI_ISL_19256195 | IIb A.2 | 10/5/2022 |  | - | - |
| 62 |  | EPI_ISL_19256196 | IIb A.2.2 | 9/30/2022 |  | - | - |
| 63 |  | EPI_ISL_19256197 | IIb A.2 | 10/26/2022 |  | - | - |
| 64 |  | EPI_ISL_19256198 | IIb A.3 | 9/14/2022 |  | - | - |
| 65 |  | EPI_ISL_19256199 | IIb A.2.3 | 3/15/2023 |  | - | - |
| 66 |  | EPI_ISL_19256200 | IIb A.2.2 | 11/1/2022 |  | - | - |
| 67 |  | EPI_ISL_19256201 | IIb A.2.2 | 10/13/2022 |  | - | - |
| 68 |  | EPI_ISL_19256202 | IIb A.2 | 9/11/2022 |  | - | - |
| 69 |  | EPI_ISL_19256203 | IIb A.2 | 9/14/2022 |  | - | - |
| 70 |  | EPI_ISL_19256204 | IIb B.1 | 2022 |  | - | - |
| 71 |  | EPI_ISL_19256205 | IIb A.2.2 | 9/22/2022 |  | - | - |
| 72 |  | EPI_ISL_19256206 | IIb A.2.3 | 9/23/2022 |  | - | - |
| 73 |  | EPI_ISL_19256207 | IIb A.2 | 9/18/2022 |  | - | - |
| 74 |  | EPI_ISL_19256208 | IIb A.2.3 | 9/13/2022 |  | - | - |
| 75 |  | EPI_ISL_19256209 | IIb B.1 | 9/21/2022 |  | - | - |
| 76 |  | EPI_ISL_19256210 | IIb A.2.2 | 9/16/2022 |  | - | - |
| 77 |  | EPI_ISL_19256211 | IIb A.2.2 | 9/12/2022 |  | - | - |
| 78 |  | EPI_ISL_19256212 | IIb A.2.2 | 9/30/2022 |  | - | - |
| 79 |  | EPI_ISL_19256213 | IIb A.2.2 | 9/23/2022 |  | - | - |
| 80 |  | EPI_ISL_19256214 | IIb A.2.2 | 9/29/2022 |  | - | - |
| 81 |  | EPI_ISL_19256215 | IIb A.2.2 | 9/16/2022 |  | - | - |
| 82 |  | EPI_ISL_19256216 | IIb A.2 | 9/20/2022 |  | - | - |
| 83 |  | EPI_ISL_19256217 | IIb A.2 | 9/15/2022 |  | - | - |
| 84 |  | EPI_ISL_19256218 | IIb B.1 | 9/25/2022 |  | - | - |
| 85 |  | EPI_ISL_19256219 | IIb A.2.2 | 11/2/2022 |  | - | - |
| 86 |  | EPI_ISL_19256220 | IIb A.2 | 10/30/2022 |  | - | - |
| 87 |  | EPI_ISL_19256221 | IIb A.2.3 | 10/24/2022 |  | - | - |
| 88 |  | EPI_ISL_19256222 | IIb A.2.3 | 9/17/2022 |  | - | - |
| 89 |  | EPI_ISL_19256223 | IIb A.2 | 10/26/2022 |  | - | - |
| 90 |  | EPI_ISL_19256224 | IIb A.2.3 | 10/24/2022 |  | - | - |
| 91 |  | EPI_ISL_19256225 | IIb A.2.2 | 10/14/2022 |  | - | - |
| 92 |  | EPI_ISL_19256226 | IIb A.2.3 | 10/24/2022 |  | - | - |
| 93 |  | EPI_ISL_19256227 | IIb A.2.3 | 4/17/2023 |  | - | - |
| 94 |  | EPI_ISL_19256228 | IIb A.2.3 | 10/17/2022 |  | - | - |
| 95 |  | EPI_ISL_19256229 | IIb A.2.3 | 9/17/2022 |  | - | - |
| 96 |  | EPI_ISL_19256230 | IIb A.2.3 | 9/20/2022 |  | - | - |
| 97 |  | EPI_ISL_19256231 | IIb A.3 | 2022 |  | - | - |
| 98 |  | EPI_ISL_19256232 | IIb A.3 | 10/11/2022 |  | - | - |
| 99 |  | EPI_ISL_19256233 | IIb A.2.3 | 9/13/2022 |  | - | - |
| 100 |  | EPI_ISL_19256234 | IIb A.2 | 10/8/2022 |  | - | - |
| 101 |  | EPI_ISL_19256235 | IIb A.2.3 | 10/5/2022 |  | - | - |
| 102 |  | EPI_ISL_19256236 | IIb A.2.3 | 3/21/2023 |  | - | - |
| 103 |  | EPI_ISL_19256237 | IIb A.2.3 | 2/21/2023 |  | - | - |
| 104 |  | EPI_ISL_19256238 | IIb A.2.2 | 2022 |  | - | - |
| 105 |  | EPI_ISL_19256239 | IIb A.2.3 | 2022 |  | - | - |
| 106 |  | EPI_ISL_19256240 | IIb A.2.3 | 11/1/2022 |  | - | - |
| 107 |  | EPI_ISL_19256241 | IIb A.2.2 | 2022 |  | - | - |
| 108 |  | EPI_ISL_19256242 | IIb A.2.3 | 10/1/2022 |  | - | - |
| 109 |  | EPI_ISL_19256243 | IIb A.3 | 10/26/2022 |  | - | - |
| 110 |  | EPI_ISL_19256244 | IIb A.2.3 | 2022 |  | - | - |
| 111 |  | EPI_ISL_19256245 | IIb A.3 | 2022 |  | - | - |
| 112 |  | EPI_ISL_19256246 | IIb A.3 | 2022 |  | - | - |
| 113 |  | EPI_ISL_19256247 | IIb A.2 | 8/30/2022 |  | - | - |
| 114 |  | EPI_ISL_19256248 | IIb A.2 | 8/30/2022 |  | - | - |
| 115 |  | EPI_ISL_19256249 | IIb A.2.3 | 9/16/2022 |  | - | - |
| 116 |  | EPI_ISL_19256250 | IIb A.2 | 9/16/2022 |  | - | - |
| 117 |  | EPI_ISL_19256251 | IIb A.2.3 | 2/20/2023 |  | - | - |
| 118 |  | EPI_ISL_19256252 | IIb A.2.3 | 1/1/2022 |  | - | - |
| 119 |  | EPI_ISL_19256253 | IIb A.2.3 | 1/8/2023 |  | - | - |
| 120 |  | EPI_ISL_19256254 | IIb A.2.3 | 10/24/2022 |  | - | - |
| 121 |  | EPI_ISL_19256255 | IIb A.2.3 | 10/21/2022 |  | - | - |
| 122 |  | EPI_ISL_19256256 | IIb A.2.3 | 3/14/2023 |  | - | - |
| 123 |  | EPI_ISL_19256257 | IIb A.2.3 | 9/18/2022 |  | - | - |
| 124 |  | EPI_ISL_19256258 | IIb A.2 | 9/28/2022 |  | - | - |
| 125 |  | EPI_ISL_19256259 | IIb A.2.3 | 9/19/2022 |  | - | - |
| 126 |  | EPI_ISL_19256260 | IIb A.2.3 | 10/30/2022 |  | - | - |
| 127 |  | EPI_ISL_19256261 | IIb A.2 | 10/13/2022 |  | - | - |
| 128 |  | EPI_ISL_19256262 | IIb A.2 | 9/28/2022 |  | - | - |
| 129 |  | EPI_ISL_19256263 | IIb A.2.3 | 12/26/2022 |  | - | - |
| 130 |  | EPI_ISL_19256264 | IIb A.2 | 9/26/2022 |  | - | - |
| 131 |  | EPI_ISL_19256265 | IIb A.2.3 | 10/7/2022 |  | - | - |
| 132 |  | EPI_ISL_19256266 | IIb A.2.1 | 10/12/2022 |  | - | - |
| 133 |  | EPI_ISL_19256267 | IIb A.2.3 | 10/14/2022 |  | - | - |
| 134 |  | EPI_ISL_19256268 | IIb A.2.3 | 9/27/2022 |  | - | - |
| 135 |  | EPI_ISL_19256269 | IIb A.2.3 | 9/14/2022 |  | - | - |
| 136 |  | EPI_ISL_19256270 | IIb A.2.3 | 9/12/2022 |  | - | - |
| 137 |  | EPI_ISL_19256271 | IIb A.2.3 | 9/27/2022 |  | - | - |
| 138 |  | EPI_ISL_19256272 | IIb A.2 | 9/22/2022 |  | - | - |
| 139 |  | EPI_ISL_19256273 | IIb A.2.3 | 9/27/2022 |  | - | - |
| 140 |  | EPI_ISL_19256274 | IIb A.2 | 10/8/2022 |  | - | - |
| 141 |  | EPI_ISL_19256275 | IIb A.2 | 9/27/2022 |  | - | - |
| 142 |  | EPI_ISL_19256276 | IIb | 1/1/2022 |  | - | - |
| 143 |  | EPI_ISL_19256277 | IIb A.2.3 | 2/21/2023 |  | - | - |
| 144 |  | EPI_ISL_19256278 | IIb A.2.3 | 1/8/2023 |  | - | - |
| 145 |  | EPI_ISL_19256280 | IIb A.2.3 | 3/20/2023 |  | - | - |
| 146 |  | EPI_ISL_19256281 | IIb | 9/21/2022 |  | - | - |
| 147 |  | EPI_ISL_19256282 | IIb A.2.3 | 9/19/2022 |  | - | - |
| 148 |  | EPI_ISL_19256283 | IIb A | 12/30/2022 |  | - | - |
| 149 |  | EPI_ISL_19256284 | IIb A.3 | 1/24/2023 |  | - | - |
| 150 |  | EPI_ISL_19256285 | IIb A.2.3 | 9/26/2022 |  | - | - |
| 151 |  | EPI_ISL_19256286 | IIb A | 1/1/2023 |  | - | - |
| 152 |  | EPI_ISL_19256287 | IIb A.2.3 | 9/25/2022 |  | - | - |
| 153 |  | EPI_ISL_19256288 | IIb A.2.3 | 1/2/2023 |  | - | - |
| 154 |  | EPI_ISL_19256289 | IIb A.2.3 | 9/26/2022 |  | - | - |
| 155 |  | EPI_ISL_19256290 | IIb A.2.3 | 9/30/2022 |  | - | - |
| 156 |  | EPI_ISL_19256291 | IIb A.2 | 9/26/2022 |  | - | - |
| 157 |  | EPI_ISL_19256292 | IIb A.2.2 | 5/31/2023 |  | - | - |
| 158 |  | EPI_ISL_19256293 | IIb A.2.3 | 9/23/2022 |  | - | - |
| 159 |  | EPI_ISL_19256294 | IIb A.2.2 | 5/31/2023 |  | - | - |
| 160 |  | EPI_ISL_19256295 | IIb A.3 | 10/2/2022 |  | - | - |
| 161 | Cameroon | EPI_ISL_19256296 | IIb | 2022 |  | - | - |
| 162 |  | EPI_ISL_19256297 | IIb | 2022 |  | - | - |
| 163 |  | EPI_ISL_19256298 | IIb | 2022 |  | - | - |
| 164 |  | EPI_ISL_19256299 | IIb | 10/13/2022 |  | - | - |
| 165 |  | EPI_ISL_19256300 | IIb | 10/27/2022 |  | - | - |
| 166 |  | EPI_ISL_19256301 | IIb | 11/8/2022 |  | - | - |
| 167 |  | EPI_ISL_19256303 | IIb | 11/11/2022 |  | - | - |
| 168 |  | EPI_ISL_19256304 | IIb | 7/22/2021 |  | - | - |
