# Supplementary figures and images for: Tracking the evolutionary footprint of Mpox in West Africa: phylogenetic and clade analysis
Source: Epidemiol Infect. 2025 Oct 7;154:e46. doi: 10.1017/S0950268825100411 (PMC13100924; doi:10.1017/S0950268825100411)

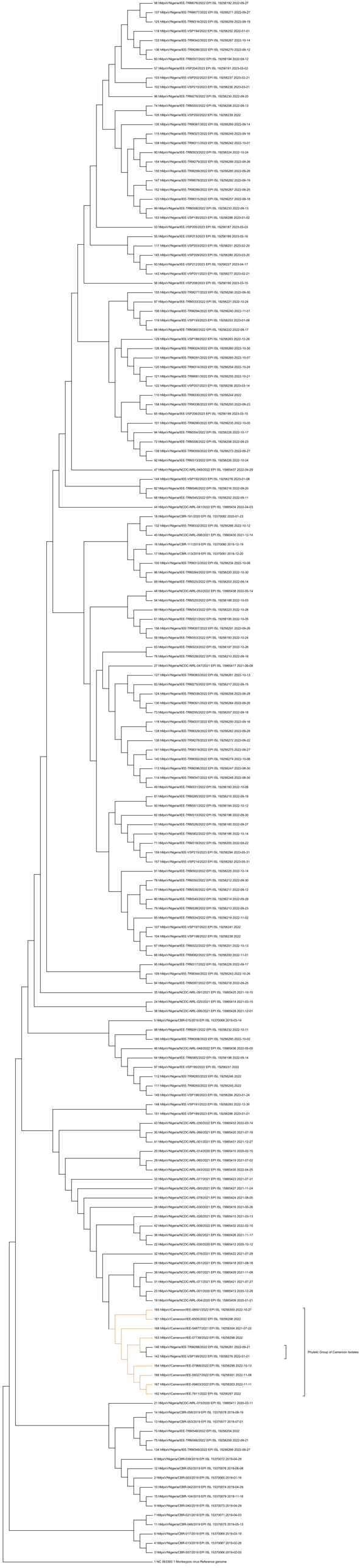

Supplement: Oladipo et al. supplementary material [file S0950268825100411sup001.zip › S0950268825100411sup002.pdf]
